# Type 3 immune response protects against *Salmonella* Typhimurium infection in the small intestine of neonatal rats

**DOI:** 10.1080/22221751.2024.2417867

**Published:** 2024-10-22

**Authors:** Zhipeng Yang, Mei Zhang, Ning Gao, Jian Peng, Hongkui Wei

**Affiliations:** a Department of Animal Nutrition and Feed Science, College of Animal Science and Technology, Huazhong Agricultural University, Wuhan, People’s Republic of China; b The Cooperative Innovation Center for Sustainable Pig Production, Wuhan, People’s Republic of China; c Frontiers Science Center for Animal Breeding and Sustainable Production, Wuhan, People’s Republic of China; d Hubei Hongshan Laboratory, Wuhan, People’s Republic of China

**Keywords:** Immunity, IL-17, RORγt, pathogen, Cintirorgon

## Abstract

Bacterial infections, particularly *Salmonella*, pose a significant health risk to neonates due to their underdeveloped immune systems. Understanding the immune responses in the neonatal intestine during *S.* Typhimurium infection is crucial for developing effective therapeutic and prevention strategies. This study found neonatal rats exhibited severe symptoms, including significant mortality, body weight loss, diarrhea, and bacterial load increases in the gastrointestinal tract and various organs, particularly in the ileum. Moreover, neonatal rats exhibited a high percentage of type 3 immune cells including Th17, γδT17, and ILC3 after *S.* Typhimurium infection. Furthermore, cintirorgon treatment during early life, the agonist of RORγt, significantly enhanced IL-17A-secreting type 3 immune response and alleviated the symptoms. Our data reveal targeting RORγt and IL-17A pathways may offer a promising therapeutic strategy for bacterial infections in neonatal populations.

## Introduction

Bacterial infections, particularly in neonates, pose a significant global health burden, especially in low-income and middle-income countries [1,2]. The immune system of newborns is relatively underdeveloped, making them more vulnerable to pathogenic infections [3]. Understanding the immune response in the intestine of newborns during infection is essential for developing effective prevention and therapeutic strategies.

Bacterial infections, particularly *Salmonella,* cause substantial infant morbidity and mortality [4]. Serotype Typhimurium causes the largest proportion of *Salmonella* infections in infants for gastroenteritis infections [4]. *Salmonella* Typhimurium (*S.* Typhimurium), is a zoonotic pathogen that causes fever, chills, diarrhea, intestinal bleeding, and sepsis in humans [5]. Previous research has mainly focused on adult models, for instance, in adult mice, acute *S.* Typhimurium infection causes the production of interferon (IFN)-γ, which mediates the delay of disease recovery [6]. However, this limits our understanding of the distinct immune responses in the developing neonatal intestine.

Type 1 and 2 immune responses are traditionally considered critical for defending against many pathogens, including *Salmonella*. Type 1 immune cells, driven by the transcription factor T-bet and producing cytokines like IFN-γ, are essential for controlling intracellular pathogens [7]. Type 2 immune cells, characterized by GATA-binding protein 3 (GATA3) and cytokines such as Interleukin (IL)-4, play a significant role in defending against extracellular parasites and mediating allergic responses [8]. However, the interplay between these immune pathways is complex, with emerging evidence suggesting that type 3 immunity also plays a significant role in host defense against *Salmonella*. Type 3 immunity, is characterized by the key transcription factor retinoic acid-related orphan receptor (RoRγt) and the expression of IL-17A, IL-17F, and IL-22 [9], mainly facing the extracellular bacterial infection and maintaining intestinal epithelial homeostasis [10,11]. It has been demonstrated that the number of RORγt^+^ cells gradually increased after mice birth implying that type 3 immunity serves a crucial role in the intestine [12]. Whereas the role of type 3 immunity in *S.* Typhimurium infection in neonates has not been cleared.

Therefore, our study aimed to investigate the immune response in the intestine of neonatal rats during *S.* Typhimurium infection. By establishing a neonatal infection model, we observed the infection rule in the neonatal rats and the activation of 3 types of immune response. Additionally, we utilized RORγt agonists to further elucidate the function of type 3 immunity underlying *S.* Typhimurium infection. The findings of this study provide insights into potential prevention targets for enhancing the immune response in neonatal hosts. This research holds promise for the development of more effective strategies to combat bacterial infections in this vulnerable population.

## Materials and methods

### Animals

The present study used Wistar rats obtained from the Animal Experiment Center at Hubei Disease Prevention and Control Center (Wuhan, China). The animal welfare and experimental procedures were carried out following the relevant ethical regulations of Huazhong Agricultural University (ID: 202401050001). The rats were housed in a specific pathogen-free air-conditioned room at 23 ± 2 °C, with food and water provided ad libitum.

### Experimental design

**For experiment 1**

To determine the accurate infectious bacterial dose, we orally administered 3 bacterial doses (1 × 10^8^, 5 × 10^8^, 2.5 × 10^9^ CFU) *S.* Typhimurium (strain SL1344) in the 14-day-old rat and observed the survival rate and body weight variation in 72 hours. Next, 5 pregnant Wistar rats were selected and monitored daily until parturition. After birth, the size of each litter was reduced to 10 pups. All pups were orally administered 5 × 10^8^ CFU *S.* Typhimurium on day 14 after birth. 1 litter was used to observe the survival rate. Randomly, 2 rats from each litter were selected and asphyxiated by CO_2_ inhalation and sacrificed at 0 , 6 , 24 , 48 , and 72 h post-infection.

**For experiment 2**

Three pregnant Wistar rats were selected and monitored daily until parturition. After birth, the size of each litter was reduced to 8–10 pups. Rats in the *S.* Typhimurium groups were fed with PBS from day 1 to day 13 every two days. Rats in the Cintirorgon + *S.* Typhimurium groups were fed with 10 mg/Kg body weight Cintirorgon (MCE, Cat# HY-104037) once every two days. All pups were orally administered 5 × 10^8^ CFU *S.* Typhimurium (strain SL1344) on day 14 after birth. 2 rats from every litter from two groups were randomly chosen and were asphyxiated by CO_2_ inhalation and sacrificed. The rest of the rats recorded the probability of survival and body weight variation.

### Sample collection

Colon, jejunum, ileum, mesenteric lymph node (MLN), liver, spleen, and kidney tissues were collected from all rats and fixed in 4% paraformaldehyde fix solution and were collected and stored at – 80 ℃ for RNA and protein isolation.

### Organ index

The isolated rat liver, kidney, and spleen were weighed on an analytical balance, and the calculation formula was as follows:

Organindex(g/kg)=(organweight(g))/(bodyweight(kg))

### Diarrhea index

Observe the feces of rats every day and evaluate them according to the following scoring criteria.


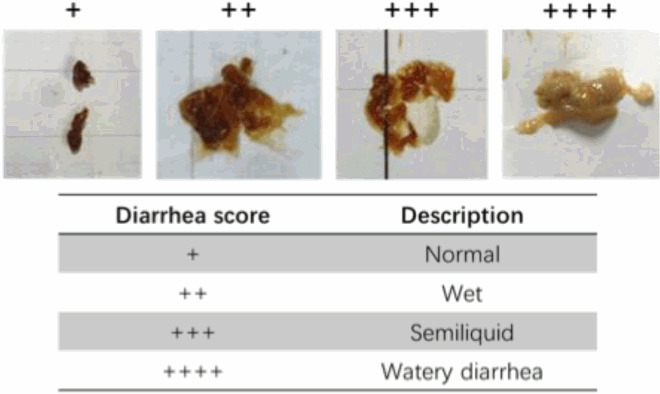


### Bacterial load assay

The bacterial load in different organs is quantitatively determined as follows: approximately 100 mg of the sample is resuspended in 1 mL of sterile phosphate-buffered saline (PBS), which is recorded as the 10^−1^ dilution. Then, 20 μL of the 10^−1^ dilution is added to 200 μL of PBS, creating the 10^−2^ dilution. Next, the sample is serially diluted and plated onto Bismuth Sulfite Agar (Hopebiol, Qingdao, China) using the dilution method. After incubation for 12–16 hours, the colony-forming units (CFUs) are counted.

### Hematoxylin and eosin (H&E) staining

First, deparaffinize and hydrate to water. Next, stain in hematoxylin solution: immerse the slides in hematoxylin solution for 3–5 minutes and rinse them in water. Then, differentiate the sections with acid alcohol, and rinse again. Blue up the sections with ammonia solution, and wash in slowly running tap water. After that, stain in eosin. Finally, dehydrate and mount. The section was scanned with Pannoramic SCAN. The morphology and histological Score were analyzed by CaseViewer software. For each sample, at least 6 intact and well-oriented crypts were measured as the distance from the apical side to the basal side of the crypt.

### Immunofluorescence staining

Tissue sections were washed three times with PBS and blocked with 5% BSA for 1 hour at room temperature. The sections were then incubated with primary antibody (anti-MPO, 1:100 in PBS) (Abclonal, Wuhan, China) overnight at 4°C. The next day, the primary antibody was removed, and the sections were washed three times with PBS. Fluorescent secondary antibody (1:150 in PBS) was added and incubated for 30 minutes at room temperature in the dark. After washing three times with PBS, DAPI staining solution (200 μL) was applied for 20 minutes at room temperature in the dark. The sections were washed three more times with PBS, mounted with 5 μL anti-quenching solution, covered with coverslips, and stored at 4°C in the dark. Finally, the slides were observed and photographed using a laser confocal microscope.

### RNA isolation and quantitative real-time PCR

Total RNA was extracted using the TRIzol reagent (Vazyme, Nanjin, China). The concentration of the RNA was then detected using a NanoDrop ND-1000 Spectrophotometer. The RNA was transcribed into cDNA using Reverse Transcriptase (Abclonal, Wuhan, China). Quantification of the mRNA levels was performed using quantitative real-time (qPCR) on a real-time PCR system. The SYBR Green qPCR Master Mix (Abclonal, Wuhan, China) was used. The sequences of the primers are listed in Table 1. The mean of the triplicate cycle thresholds (Ct) of the target gene was normalized to the mean of the triplicate Ct of the reference β-actin gene using the formula “2-ΔΔCt”, yielding the relative gene expression level values.

**Table 1.** Primers used for RT-PCR.

Gene	Primers sequence (5′−3′)	Size (bp)	Accession no.
β-actin	F: GCAGGAGTACGATGAGTCCG R: ACGCAGCTCAGTAACAGTCC	74	NM_031144.3
IL-17A	F: GTGAAGGCAGCGGTACTCAT R: GGGTGAAGTGGAACGGTTGA	162	NM_001106897.1
IL-22	F: ATACATCGTCAACCGCACCT R: TGTAAGGCTGGAATCTGTCTGA	194	NM_001191988.1
RORγt	F: GCAAAGAAGACCCACACCTCAC R: GCCGAACTTGACAGCATCTCT	261	NM_001427272.2
T-bet	F: AATGACGGTGAGCCAGAGG R: GTAGGCAGTCACGGCAATG	87	NM_001107043.1
GATA3	F: CTCCAGTCCGCATCTCTTCA R: ACCTGATACTTGAGGCACTCTT	129	NM_133293.2
IFNγ	F: TGGATGCTATGGAAGGAAAGAG R: CTGTTGCTGAAGAAGTTAGTGA	196	NM_138880.3
IL-4	F: CAAGGAACACCACGGAGAACGA R: AGACCGCTGACACCTCTACAGA	148	NM_201270.1
IL-13	F: CTCGCTTGCCTTGGTGGTCTTG R: GCTGTCAGGTCCACGCTCCATA	161	NM_053828.1
IL-1β	F: CACCTCTCAAGCAGAGCACAG R: GGGTTCCATGGTGAAGTCAAC	79	NM_031512.2
IL-23	F: GCAGCGTTCTCTTCTCCGTTCC R: CGTTGGCACTAAGGGCTCAGTC	115	NM_130410.2
Claudin-1	F: GCTGTCATCGGGGGCATAAT R: CCTGGCCAAATTCATACCTGG	136	NM_031699.3
Occludin	F: ACTATGAAACCGACTACACGA R: TGATAGGTGGATATTCCCTGAG	80	NM_031329.3
ZO-1	F: CGGATGGTGCTACAAGTGATG R: CGCCTTCTGTATCTGTGTCTTC	138	NM_001106266.1

### Isolation of intestinal LPLs and flow cytometry

The isolation of intestinal LPLs was performed as previously described with probable modification [13,14]. Briefly, the small intestine was dissected, and fat and mesentery tissues were removed. Intestines were dissected longitudinally and subsequently cut into several pieces, followed by washing with cold Hanks’ Balanced Salt Solution. To remove epithelial cells, the intestines were then incubated successively with 2% FBS, 1 mM dithiothreitol, 30 mM EDTA-PBS once for 30 min at 37 °C while shaking at 200 rpm. The tissues were then digested with 50 μg/mL DNase I and 300 U/mL collagenase VIII in RPMI1640 medium for 30 min at 37 °C while shaking at 200 rpm. The digested tissues were homogenized by vigorous shaking and filtered with a 70 µm cell strainer. Mononuclear cells were then harvested from the interphase of an 80 and 40% Percoll gradient after spinning at 800×g for 15 min at RT.

For cytokine staining, cells were stimulated with Cell Stimulation Cocktail (plus protein transport inhibitors) (500X) (eBioscience, Cat#: 00-4975-93) for 5 h. The panel design refers to ref [15], the detailed antibodies used are shown in Table 2. Analysis was performed on a CytoflexLX (Beckman, USA).

**Table 2.** Antibody used for flow.

Marker	Channel	Cat#	Company
SIRP-α	PE	MA517504	Thermo
CD3	PE	550353	BD Pharmingen
CD45RA	PE	554881	BD Pharmingen
CD127	Alexa Fluor594	FAB8484T	R&D system
CD45	APC-Cy7	561586	BD Pharmingen
RORγt	APC	130-123-840	Miltenyi
IL-22	PerCP/Cyanine5.5	516411	BD Pharmingen
IL-17	PE-Cy7	25-7177-82	Thermo
CD3	FITC	559975	BD Pharmingen
γδ T	BV650	745392	BD Pharmingen
CD4	APC	550057	BD Pharmingen

### Statistical analysis

We analyzed the data using GraphPad Prism (v 9.0.0), Microsoft Excel (2023), and FlowJo (v 10.8.1), with results expressed as the means ± standard error of the mean (SEM). Firstly, the data were assessed for normality of distribution using the Shapiro−Wilk test. For data with a normal distribution, statistically significant differences at different time points were determined using one-way ANOVA followed by Tukey's post hoc multiple comparison tests. For data with a non-normal distribution, statistically significant differences at different time points were determined using the Kruskal–Wallis test followed by Dunn's multiple comparisons tests. For comparisons between two groups, a t-test was used for normally distributed data, while the Mann−Whitney U-test was used for non-normally distributed data. Statistical significance was defined as *P* < 0.05. A *p*-value of less than 0.05 was considered statistically significant.

## Results

### Neonatal rats showed severe symptoms after *S.* Typhimurium infection

**Figure 1.**
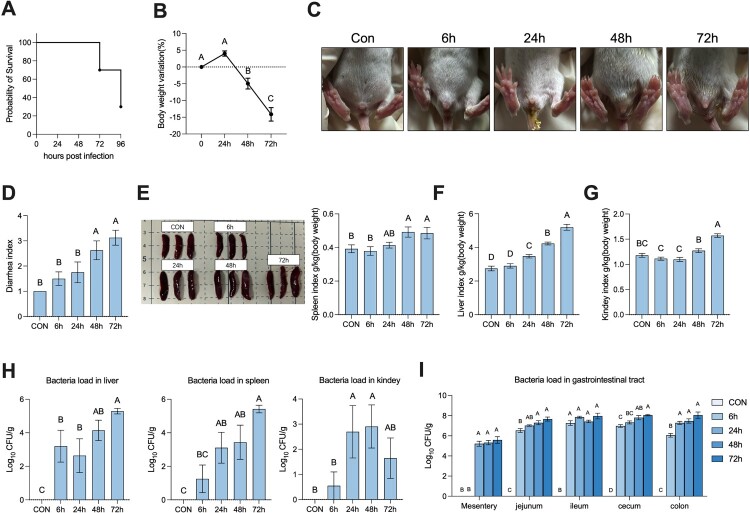
Neonatal rats showed severe symptoms after *S.* Typhimurium infection. (A) Percent survival from 0 hours post-infection to 96 hours post-infection. (B) Body weight variation from 0 hours post-infection to 72 hours post-infection. (C) Representative diarrhea images from 0 hours post-infection to 72 hours post-infection. (D) Diarrhea variation from 0 hours post-infection to 72 hours post-infection. (E) Representative spleen images and spleen index from 0 hours post-infection to 72 hours post-infection. (F&G) Liver and Kidney index from 0 hours post-infection to 72 hours post-infection. (H) Liver, spleen, and kidney bacterial load from 0 hours post-infection to 72 hours post-infection. (I) MLN, jejunum, ileum, cecum, and colon bacterial load from 0 hours post-infection to 72 hours post-infection. Values are expressed as means ± SEM, n = 8. Different letters represent significant differences (*p* < 0.05).

Firstly, we tested three doses of *S*. Typhimurium – 1 × 10⁸ CFU, 5 × 10⁸ CFU, and 2.5 × 10⁹ CFU. We observed that the low dose had a mild effect, while the high dose induced a significant response, leading to the death of all rats by the 72 hours post challenge. Based on these findings, we ultimately selected a moderate dose for our next experiments (Supplementary Figure 1). Next, we orally administrated 5 × 10^8^ CFU *S.* Typhimurium in the 14-day rats to establish an *S.* Typhimurium infection model. We found that rats began to die at 72 hours post-infection, and all were dead at 120 hours post-infection (Figure 1A). The body weight of the rats showed a significant decrease at 48 hours post-infection and continued to decrease at 72 hours post-infection (Figure 1B). Next, we evaluated the diarrhea status of the rats and found that rats began to show severe diarrhea at 48 hours post-infection (Figure 1C, D). Moreover, all the organs, including the spleen, liver, and kidney, showed enlargement after 48 hours post-infection (Figure 1E-G). We also detected *S.* Typhimurium loading in the organs and various intestinal segments by plate counting and found that *S.* Typhimurium began loading in the organs after 6 hours post-infection, with total samples loaded in the liver and spleen after 72 hours post-infection, whereas some samples were not loaded in the kidney even at 72 hours post-infection (Figure 1H). Furthermore, the *S.* Typhimurium loading in the MLN occurred after 24 hours post-infection. The *S.* Typhimurium loading in the jejunum, cecum, and colon gradually increased after infection, and was at a similar level, whereas the ileum showed a high level of *S.* Typhimurium loading from 6 to 72 hours post-infection (Figure 1I). Collectively, we observed symptoms such as death, weight loss, severe diarrhea, and organ enlargement in the *S.* Typhimurium infection model in neonatal rats. In addition, *S.* Typhimurium loading in various organs and intestinal segments was also detected.

### Neonatal rats showed severe intestinal damage after *S.* Typhimurium infection

**Figure 2.**
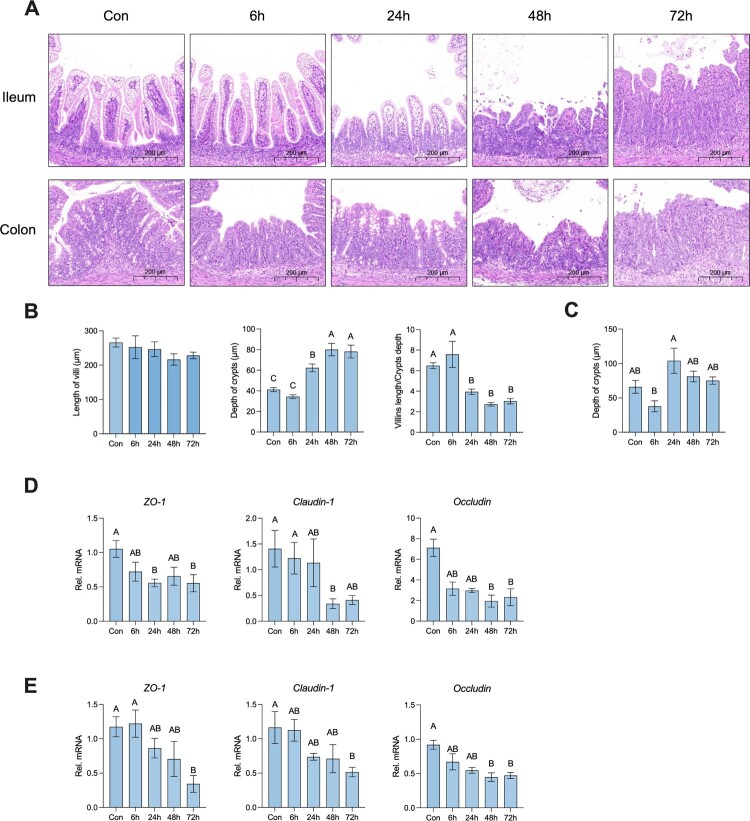
Neonatal rats showed severe intestinal damage after *S.* Typhimurium infection. (A) Representative image of ileum and colon morphology (the ruler is 200μm). (B) Statistical analysis of villi length, crypt depth and ratio of villi length and crypt depth of ileum. (C) Statistical analysis of crypt depth of ileum. (D) The relative mRNA expression of the gene for ZO-1, Cluadin-1, and Occcludin of ileum. (E) The relative mRNA expression of the gene for ZO-1, Cluadin-1, and Occcludin of colon. Values are expressed as means ± SEM, n = 8. Different letters represent significant differences (*p* < 0.05).

Next, we chose the ileum and colon to observe the morphology integrity by HE staining (Figure 2A). There was no significant difference in the length of the villi after *S.* Typhimurium infection, whereas *S.* Typhimurium infection significantly induced the extension of crypt depth after 24 hours post-infection. Moreover, the ratio of villi length to crypt depth significantly decreased after 24 hours post-infection (Figure 2B). Furthermore, the colon showed an increase in crypt depth after 24 hours post-infection (Figure 2C). Tight junctions are mainly involved in the formation and maintenance of the barrier function between epithelial cells, thus we explored the change of tight junctions after *S.* Typhimurium infection. The relative mRNA expression of ZO-1, Claudin-1, and Occludin in the ileum and colon was significantly decreased after *S.* Typhimurium infection, especially 72 hours post-infection (Figure 2D, E). collectively, *S.* Typhimurium infection induced severe intestinal damage both in the ileum and colon of neonatal rats.

### Neonatal rats showed transcriptional activation of type 3 immunity after *S.* Typhimurium infection

**Figure 3.**
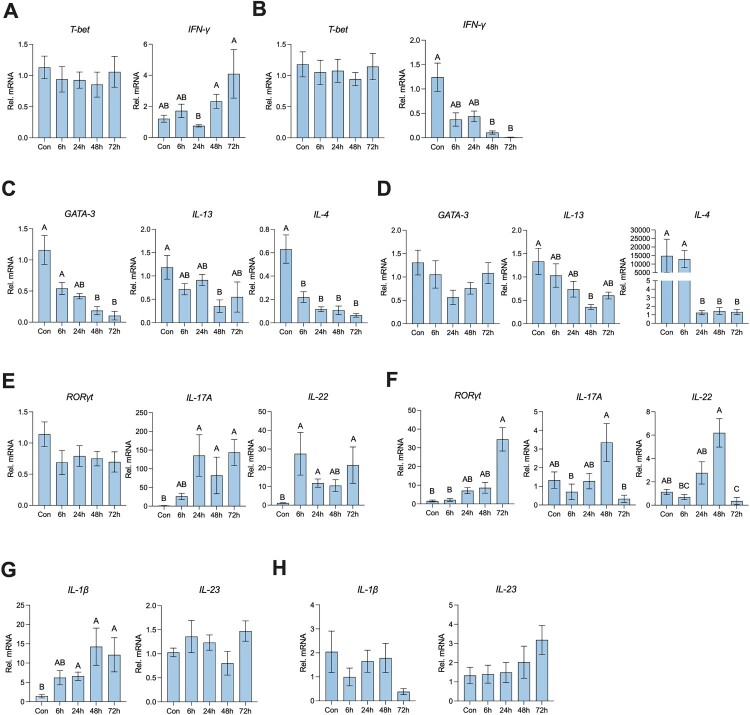
Neonatal rats showed transcriptional activation of type 3 immunity after *S.* Typhimurium infection. (A) The relative mRNA expression of the gene for *T-bet* and *IFN-γ* of the ileum and colon (B). (C) The relative mRNA expression of the gene for *GATA-3, IL-13,* and *IL-4* of the ileum and colon (D). (E) The relative mRNA expression of the gene for *RORγt, IL-17A,* and *IL-22* of the ileum and colon (F). (G) The relative mRNA expression of the gene for *IL-1β,* and *IL-23* of the ileum and colon (H). Values are expressed as means ± SEM, n = 8. Different letters represent significant differences (*p* < 0.05).

Three types of immune responses face microbial infection including bacteria, fungi, and parasites, thus we first detected the transcriptional activation of these three types of immune responses in the ileum and colon. As for type 1 immunity, we did not observe any change in *T-bet* expression in the ileum and colon. Further, the *IFN-γ* level increased in the ileum and decreased in the colon after *S.* Typhimurium infection (Figure 3A, B). As for type 2 immunity, we found that the *GATA-3* expression decreased in the ileum, while there was no significant difference in the colon. Moreover, the level of characterizing cytokines of type 2 immunity, *IL-13*, and *IL-4*, significantly decreased after *S.* Typhimurium infection in both the ileum and colon (Figure 3C, D). As for type 3 immunity, we found that *RORγt* expression increased in the colon, while there was no significant difference in the ileum. Furthermore, the transcript levels of *IL-17A* and *IL-22* were both elevated by *S.* Typhimurium infection in the ileum and colon (Figure 3E, F). Type 3 immune response induced by cytokines *IL-1β* and *IL-23* [16], thus we detected the cytokines expression in the ileum and colon. And found the expression of *IL-1β* in the ileum was elevated after 24 hours post-infection, whereas there was no significant change in the *IL-23* level (Figure 3G). And there was no significant change in the *IL-1β* and *IL-23* levels in the colon after *S.* Typhimurium (Figure 3H)*.* In summary, *S.* Typhimurium infection induced a type 3 immune transcriptional response, especially in the ileum.

### Neonatal rats showed a type 3 immune response after *S.* Typhimurium infection

**Figure 4.**
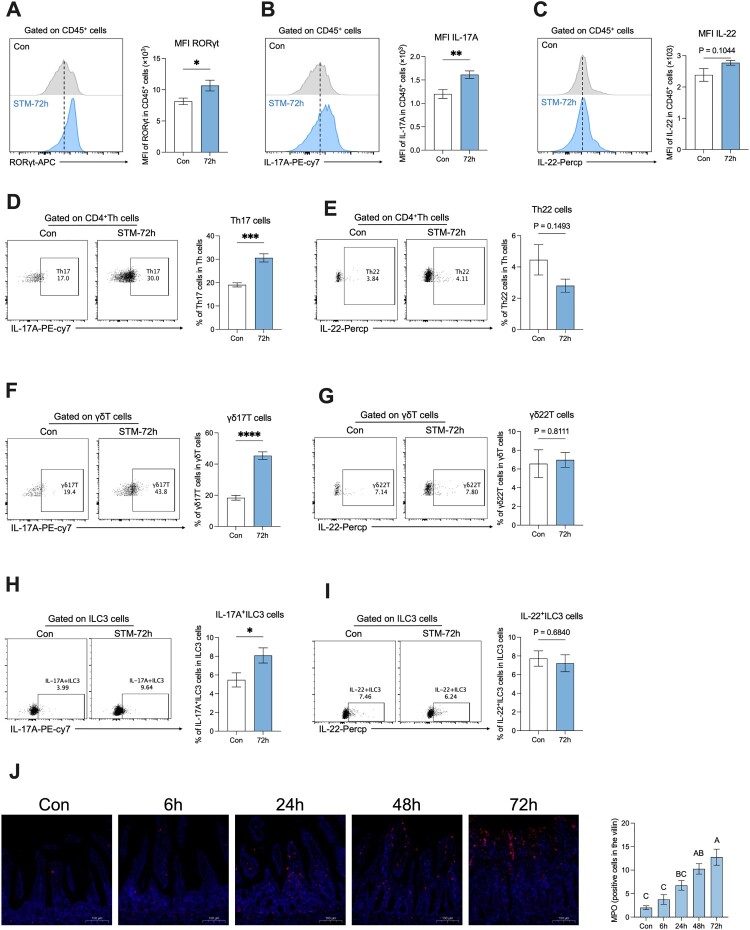
Neonatal rats showed a type 3 immune response after *S.* Typhimurium infection. (A) Representative FACS plots and MFI of RORγt, IL-17A (B), and IL-22 (C) expression in CD45^+^ cells of small intestine LPLs of rats at 72 hours post-infection. (D) Representative FACS plots and % of Th17 and Th22 (E) in small intestine LPLs of rats. (F) Representative FACS plots and % of γδT17 and γδT22 (G) in small intestine LPLs of rats at 72 hours post-infection. (H) Representative FACS plots and % of IL-17A ^+ ^ILC3 and IL-22 ^+ ^ILC3 (I) in small intestine LPLs of rats at 72 hours post-infection. (J) Representative MPO expression and MPO-positive cells in the villi from 0 hours post-infection to 72 hours post-infection. Values are expressed as means ± SEM, n = 8. Different letters represent significant differences (*p* < 0.05). *p < 0.05; **p < 0.01; ***p < 0.001; ****p < 0.0001.

To understand the detailed variation of type 3 immune cells, we first detected the expression of RORγt, IL-17A, and IL-22 in the CD45^+^ cells in the LPLs of rats. Results showed a significant increase in the MFI of RORγt in CD45^+^ cells (Figure 4A), further, the MFI of IL-17 in CD45^+^ cells was also increased after *S.* Typhimurium infection (Figure 4B), whereas there was no significant difference in the MFI of IL-22 in CD45^+^ cells (Figure 4C). We next investigated the proportion of main type 3 immune cells including Th17, γδT17, and ILC3. We found that the proportion of Th17 cells and MFI of IL-17A in the Th cells increased after *S.* Typhimurium infection (Figure 4D), while the proportion of Th22 cells did not change (Figure 4E). Similarly, the proportion of γδ17 T cells increased after *S.* Typhimurium infection (Figure 4F), while the proportion of γδT22 cells did not change (Figure 4G). Furthermore, there was a significant increase in the proportion of IL-17A ^+ ^ILC3 cells (Figure 4H), and there was no significant difference in the proportion of IL-22 ^+ ^ILC3 cells (Figure 4I). Considering the elevated IL-17A level, we next detected the MPO level in the ileum, because IL-17A promotes neutrophil recruitment to restrict the colonization of bacterial pathogen [17,18]. Results showed that the MPO-positive cells gradually increased after infection, significantly increased at 48 hours post-infection, and reached the top at 72 hours post-infection (Figure 4J). In summary, our results revealed a significant increase in the expression and proportion of type 3 immune cells, along with elevated IL-17A levels and increased MPO-positive cells following *S.* Typhimurium infection.

### Early life administration of RORγt agonist (Cintirorgon) ameliorates the severity of *S.* Typhimurium infection

Based on our previous results, we considered whether activating the type 3 immune cells response by RORγt agonist could protect against *S.* Typhimurium infection in the early life of rats. Cintirorgon has been identified as an orally bioavailable RORγt agonist and was used to activate RORγ^+^ T cells in the tumour microenvironment and render immune infiltrates more effective at countering tumour growth [19,20].

**Figure 5.**
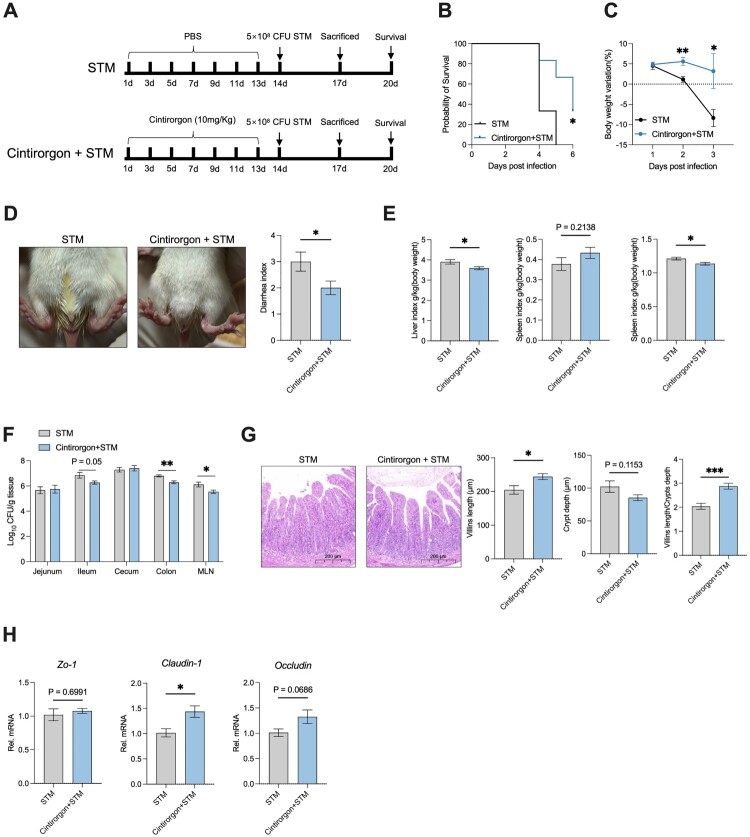
Early life administration of RORγt Agonist (Cintirorgon) ameliorates the severity of *S.* Typhimurium infection. (A) Experimental design. (B) Percent survival of rats. (C) Body weight variation of rats. (D) Representative diarrhea images and diarrhea index of rats at 72 hours post-infection. (E) liver, spleen, and kidney index of rats at 72 hours post-infection. (F) jejunum, ileum, cecum, colon, and MLN bacterial load of rats at 72 hours post-infection. (G) Representative image of ileum morphology (the ruler is 200μm) and statistical analysis of villi length, crypt depth and ratio of villi length and crypt depth of ileum. (H) The relative mRNA expression of the gene for *ZO-1*, *Cluadin-1*, and *Occludin* of ileum. Values are expressed as means ± SEM, n = 6. *p < 0.05; **p < 0.01; ***p < 0.001.

We next examined the effect of early life administration of Cintirorgon on *S.* Typhimurium-infected rats (Figure 5A). We found administration of Cintirorgon significantly alleviated the mortality rate of rats (Figure 5B). And the body weight loss of Cintirorgon treated rats was slower compared with PBS treated rats (Figure 5C). Further, we also observed diarrhea index and found administration of Cintirorgon significantly alleviated the diarrhea index of rats (Figure 5D). The organ index was also determined, and the liver and kidney index were decreased by administration of Cintirorgon (Figure 5E). We next detected the bacterial load in the gastrointestinal tract, results showed that administration of Cintirorgon suppressed the *S.* Typhimurium load in the ileum, colon, and MLN (Figure 5F). Furthermore, the morphology integrity of ileum was also detected, administration of Cintirorgon significantly prevented the shortage of villins length and promoted the villins length/Crypts depth of ileum after *S.* Typhimurium infection, while had not trend to alleviate the extension of crypt depth (Figure 5G). We also measured the mRNA expression of the tight junction and found the expression *of Claudin-1* was significantly upregulated and the expression of *Occludin* had a trend to increase by administration of Cintirorgon (Figure 5H). In summary, early life administration of Cintirorgon in *S.* Typhimurium-infected rats significantly reduced severe symptoms, and alleviated intestinal damage.

### Early life administration of RORγt agonist (Cintirorgon) enhanced the IL-17A-mediated type 3 immune response

**Figure 6.**
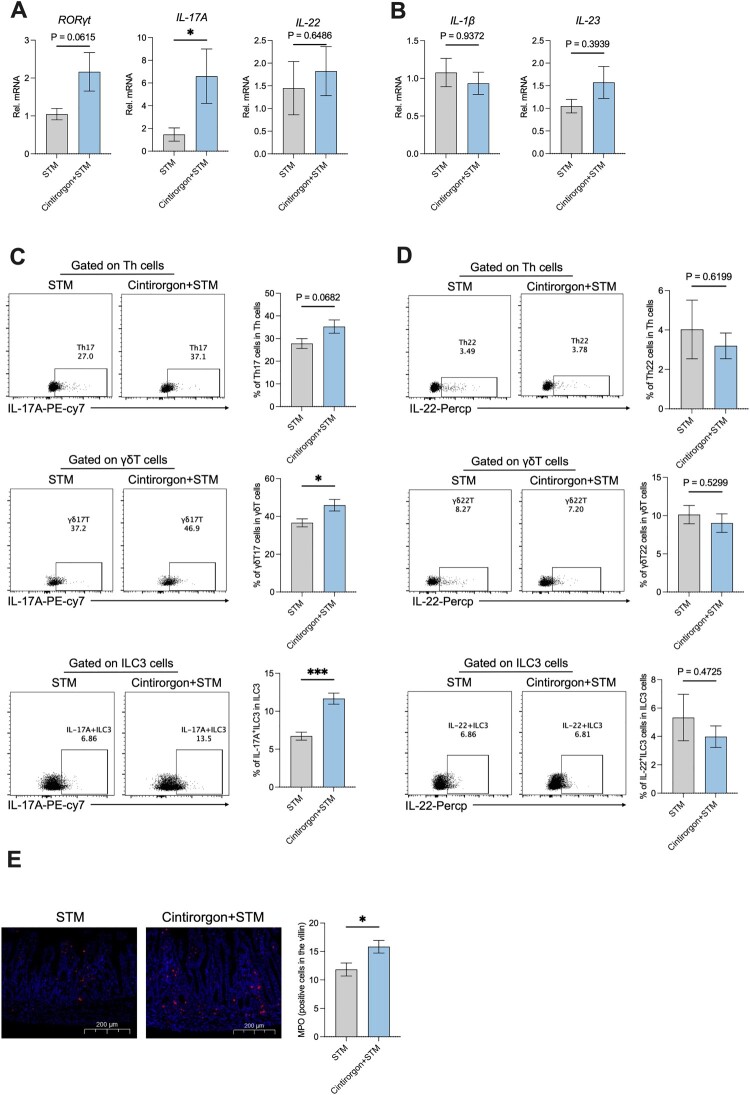
Early life administration of RORγt Agonist (Cintirorgon) enhanced the IL-17A-mediated type 3 immune response. (A) The relative mRNA expression of the gene for *RORγt*, *IL-17A*, and *IL-22* of the ileum. (B) The relative mRNA expression of the gene for *IL-1β* and *IL-23* of the ileum. (C) Representative FACS plots and % of γδT17, IL-17A ^+ ^ILC3, and Th17 in small intestine LPLs of rats at 72 hours post-infection. (D) Representative FACS plots and % of and γδT22, IL-22 ^+ ^ILC3, and Th22 in small intestine LPLs of rats at 72 hours post-infection. (E) Representative MPO expression and MPO-positive cells in the villi of rats at 72 hours post-infection. Values are expressed as means ± SEM, n = 6. *p < 0.05; ***p < 0.001.

We next explored whether activating the type 3 immune cells response by RORγt agonist could protect against *S.* Typhimurium infection in the early life of rats. We first detected the mRNA expression of type 3 immune factors and found administration of Cintirorgon had a trend to increase the mRNA expression of *RORγt* and *IL-17A* but did not change the mRNA expression of *IL-22* (Figure 6A). We next measured the type 3 immune response activated factors, *IL-1β* and *IL-23*, while there were no changes in the mRNA level of *IL-1β* and *IL-23* (Figure 6B) which indicated that administration of Cintirorgon did not affect the activated pathway of type 3 immune response. Further, we explored the percentage of immune cells in the ileum LPLs. The ratio of IL-17A^+^ ILC3 and γδT17 was significantly upregulated, and the ratio of Th17 had a trend to be increased by administration of Cintirorgon (Figure 6C). And the ratio of IL-22^+^ ILC3, γδT22, and Th22 did not change after administration of Cintirorgon (Figure 6D). Additionally, we compared the MPO-positive cells in the villi and found administration of Cintirorgon increased the MPO-positive cells in the villi (Figure 6E). Collectively, early life administration of RORγt agonist (Cintirorgon) enhanced the type 3 immune response under *S.* Typhimurium infection.

## Discussion

In this study, we established an *S.* Typhimurium infection model in neonatal rats to explore the pathogenesis and immune response to infection and investigated the potential therapeutic effects of early life administration of a RORγt agonist, Cintirorgon. Our findings highlight several key aspects of *S.* Typhimurium infection in neonatal rats, including mortality, weight loss, diarrhea, intestinal damage, and immune response alterations.

Neonatal rats exhibited severe symptoms following *S.* Typhimurium infection, including high mortality rates starting at 72 hours post-infection, significant body weight loss, and severe diarrhea. Organ enlargement was observed in the spleen, liver, and kidney at 48 hours post-infection, indicating systemic infection and organ involvement. Compared with the adult model, the intestinal microbiota does not need to be disrupted by antibiotics before *S.* Typhimurium infection [21,22], which helps investigate the crosstalk between host microbiome and infection. *S.* Typhimurium mainly colonized in the colon of adult mice and induced colitis [23]. However, the bacterial load analysis confirmed the presence of *S.* Typhimurium in various organs and intestinal segments, with the ileum showing the highest bacterial load from 6 to 72 hours post-infection. This suggests that the ileum is a primary site for bacterial colonization and persistence, and *S.* Typhimurium-infected neonatal rats may be a useful infection model of the small intestine. *S.* Typhimurium disrupts intestinal epithelial barrier integrity and promotes bacterial translocation through the effector *SpvB* [24]. Histological analysis revealed a significant intestinal change in the ileum and colon beginning 24 hours post-infection, characterized by increased crypt depth and a decreased villi length to crypt depth ratio. The intestinal epithelial cells begin to exfoliate, and the mRNA level of tight junction proteins is downregulated after 48 hours of infection. These morphological changes likely impair nutrient absorption and barrier function, contributing to the clinical symptoms of diarrhea and weight loss.

*S.* Typhimurium belongs to the intracellular bacteria, and induced IFN-γ mediated type 1 immune response [25,26]. However, previous results found that the expression of IL-17A is important for orchestrating early inflammatory responses during S. Typhimurium colitis [27]. In our study, the transcriptional activation of immune responses in the ileum and colon was compared, while type 1 and type 2 immune responses showed mixed results, type 3 immunity was markedly upregulated, as evidenced by increased RORγt expression and elevated levels of IL-17A and IL-22, and the type 3 immune response in the ileum was stronger than that in the colon. Further, MPO, the marker of neutrophil recruitment, also increased in the ileum. Consistent with the previous research in adult mice, IL-17A is required to suppress the invasion of *Salmonella Typhimurium* into enteric mucosa [28]. The cells responsible for type 3 immunity are diverse, mainly including ILC3, γδT17, and Th17 [29]. Our further analysis of the immune cell populations in the LPLs of the intestine showed an increase in IL-17A-expressing cells, including Th17, γδ17 T, and ILC3 cells. However, there was no effect on the IL-22 expression, we speculate a lack of IL-22 production in the intestine during the neonatal period is a part of the reason [30]. Collectively, these results reveal a specific activation of IL-17A-secreted type 3 immunity after *S.* Typhimurium infection in neonatal rats.

Therapeutic targeting of the type 3 immune cells pathway has been proven effective in several microbial infections [31–33]. RORγt, as an essential role in the activation of type 3 immune cells and plays a key role in the regulation of immune responses and the maintenance of immune homeostasis [34]. Our study explored whether promoting the development of type 3 immune cells after birth mitigates *S.* Typhimurium infection in neonatal rats. Early administration significantly improved survival rates, reduced body weight loss, and alleviated diarrhea. It also decreased liver and kidney enlargement, suppressed bacterial load in the gastrointestinal tract, preserved villi length, and improved villi length to crypt depth ratio. Notably, promoting the development of type 3 immune cells after birth enhanced the IL-17A-mediated type 3 immune response, as evidenced by increased expression of *Rorc* and *Il17a* mRNA, as well as a higher proportion of IL-17A^+^ ILC3, γδ17 T, and Th17 cells. The lack of significant changes in IL-22^+^ cell populations and associated mRNA expression suggests a specific augmentation of the IL-17A pathway. Additionally, the increase in MPO-positive cells in the villi indicates enhanced neutrophil activity, contributing to the antibacterial defense.

In summary, our study demonstrates that *S.* Typhimurium infection induces severe pathology and robust type 3 immune responses in neonatal rats. Promoting the development of type 3 immune cells after birth significantly ameliorates infection severity by enhancing IL-17A-mediated immune responses and preserving intestinal integrity. These findings suggest that targeting RORγt and IL-17A pathways may offer a promising therapeutic strategy for bacterial infections in neonatal populations. Further studies are warranted to explore the long-term effects and potential clinical applications of RORγt agonists in treating bacterial infections in neonates.

## Supplementary Material

Supplementary Figure 1 .docx
