# Supplementary material for: Type 3 immune response protects against Salmonella Typhimurium infection in the small intestine of neonatal rats
Source: Emerg Microbes Infect. 2024 Oct 22;13(1):2417867. doi: 10.1080/22221751.2024.2417867 (PMC11520099; doi:10.1080/22221751.2024.2417867)
Supplement: Supplementary Figure 1 .docx [file TEMI_A_2417867_SM7361.docx]

**Supplementary Figure 1 Effect of different concentrations of *S*. Typhimurium on the neonatal rats.** (A) Percent survival from 0 hours post-infection to 72 hours post-infection. (B) Body weight variation from 0 hours post-infection to 72 hours post-infection. Values are expressed as means ± SEM, n = 4. * present there is a significant difference between the CON group and 1×10^8^CFU group; # present there is a significant difference between the CON group and 5×10^8^CFU group; $ present there is a significant difference between the CON group and 1×10^8^CFU group; ***p* < 0.01; ^#^*p* < 0.001; ^##^*p* < 0.00; ^###^*p* < 0.001; ^$$$^*p* < 0.001.
